# Value of electrocardiographic left ventricular hypertrophy as a predictor of poor blood pressure control: Evidence from the China stroke primary prevention trial

**DOI:** 10.1097/MD.0000000000012966

**Published:** 2018-11-02

**Authors:** Zhenzhen Wang, Chunyan Zhang, Huihui Bao, Xiao Huang, Fangfang Fan, Yan Zhao, Juxiang Li, Jing Chen, Kui Hong, Ping Li, Yanqing Wu, Qinghua Wu, Binyan Wang, Xiping Xu, Yigang Li, Yong Huo, Xiaoshu Cheng

**Affiliations:** a Department of Cardiovascular Medicine, the Second Affiliated Hospital of Nanchang University, Nanchang; b Department of Neurology, the Second Hospital, Shanxi Medical University, Shanxi; c Department of Cardiovascular Medicine, XinHua Hospital Affiliated with Shanghai Jiao Tong University School of Medicine, Shanghai; d Department of Cardiology, Peking University First Hospital, Beijing; e National Clinical Research Study Center for Kidney Disease; State Key Laboratory for Organ Failure Research; Renal Division, Nanfang Hospital, Southern Medical University, Guangzhou, China.

**Keywords:** blood pressure control, electrocardiographic, hypertension, left ventricular hypertrophy

## Abstract

Recent studies have shown that hypertension is poorly controlled in many populations worldwide. Electrocardiographic left ventricular hypertrophy is a common manifestation of preclinical cardiovascular disease that strongly predicts cardiovascular disease morbidity and mortality. However, little information is available regarding the role of left ventricular hypertrophy in blood pressure (BP) control. We aimed to assess the relationship between electrocardiographic left ventricular hypertrophy and BP control in the China Stroke Primary Prevention Trial. The study population included 17,312 hypertensive patients who were selected from a group of 20,702 adults who had participated in the China Stroke Primary Prevention Trial and had undergone electrocardiography at baseline visit. Multivariate analysis identified left ventricular hypertrophy as a predictor of unsatisfactory BP control. The results revealed that 8.1% of hypertensive adults exhibit left ventricular hypertrophy and that the disease is more prevalent in males (12.8%) than in females. Multivariate regression analysis showed that the electrocardiographic left ventricular hypertrophy group had a significantly higher rate of unsatisfactory BP control [odds ratio (OR) 1.42, 95% confidence interval (95% CI) 1.26–1.61, *P* < .001) than the nonleft ventricular hypertrophy group.

Notable differences in BP control were also observed among males (OR 1.37, 95% CI 1.17–1.60, *P* < .001) and females (OR 1.45, 95% CI 1.18–1.77, *P* < .001) and especially among patients with comorbid diabetes (OR 2.32, 95% CI 1.31–4.12, *P* = .004). In conclusion, the results of this study indicate that electrocardiographic left ventricular hypertrophy appears to be an independent predictive factor for poor BP control, especially in females and patients with comorbid diabetes.

## 1 Introduction

Hypertension is an important worldwide public health challenge due to its high prevalence and concomitant risks of cardiovascular and kidney disease. A 2013 to 2014 nationally representative survey indicated that the prevalence of hypertension in China was 27.8%. Of those with hypertension, 31.9% were previously diagnosed; of those diagnosed, 82.9% were treated, and of those treated, only 34.6% achieved proper blood pressure (BP) control (<140/90 mm Hg); the overall control rate was only 9.7% among those with hypertension. ^[1]^ The rate of BP control was lower in females than in males and lower in rural patients than in urban patients. ^[1,2]^ Current criteria have established that the achievement of BP goals is associated with significant benefits in cardiovascular morbidity and mortality.^[3]^

Electrocardiography is a routine, accessible, cost-effective, and recommended diagnostic tool for the initial evaluation and follow-up of hypertensive patients. It is widely available, inexpensive, and carries prognostic value independent of other left ventricular hypertrophy (LVH) detection techniques (e.g., echocardiography).^[4]^ Several studies have established electrocardiographic (ECG LVH) as an independent predictor of cardiovascular morbidity and mortality.^[4–6]^ However, few studies have focused on the relationship between LVH and the achievement rates of target BP levels in patients with hypertension, as defined by the 2014 Evidence-Based Guideline for the Management of High Blood Pressure in Adults (JNC8 2014).^[7]^

The present study assessed whether LVH voltage criteria were associated with higher rates of lack of BP control in hypertensive patients during follow-up. In particular, the aim of this study was to elucidate whether LVH is associated with BP control achievement rates in a Chinese population using a large community-based sample of treated hypertensive patients. We first evaluated whether the LVH-systolic BP (SBP) control relationship could be affected by types of antihypertensive drugs or other variables, including gender, age, body mass index (BMI), and baseline SBP. We further explored other factors that influence SBP and diastolic BP (DBP) control.

## 2 Methods

### 2.1 Study Population

**Figure 1:**
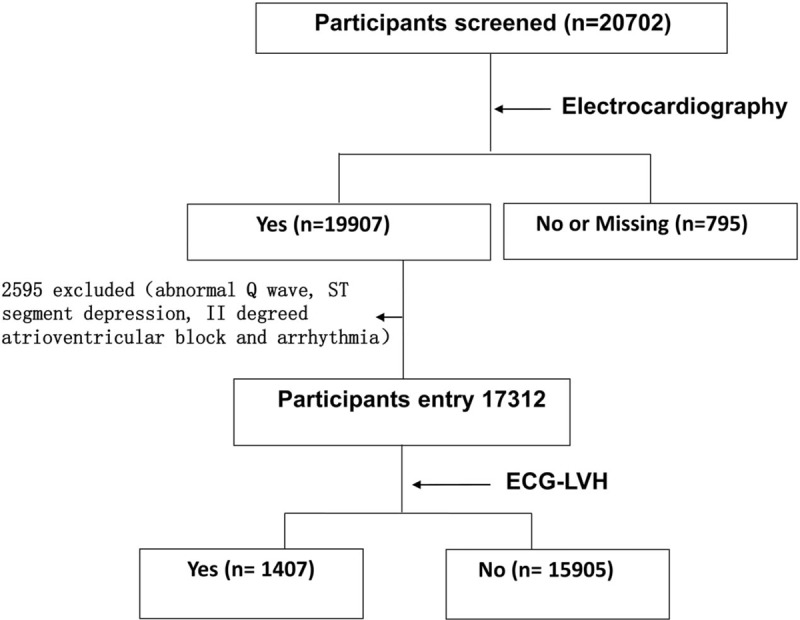
Flow of participants with electrocardiographic left ventricular hypertrophy from the China Stroke Primary Prevention Trial.

The present study population consisted of a subset of the China Stroke Primary Prevention Trial. A detailed description of the design and methodology has been previously published.^[8]^ Eligible participants included hypertensive men and women who were 45 to 75 years of age. The major exclusion criteria included a history of stroke, myocardial infarction, heart failure, coronary revascularization, and/or congenital heart disease. Individuals with missing electrocardiogram measurements, indecipherable electrocardiogram results, and/or electrocardiogram abnormalities with inconsistent changes (Q wave, ST segment depression, second degree atrioventricular block, or arrhythmia) at baseline (Fig. 1) were further excluded. A total of 17,312 participants with hypertension were selected for the present analysis.

The China Stroke Primary Prevention Trial protocol was approved by the ethics committee of the Institute of Biomedicine, Anhui Medical University, Hefei, China (FWA assurance number FWA00001263).^[8]^ All participants provided written informed consent for their participation in the study protocol. The study protocol was conducted according to the principles of the Declaration of Helsinki designed to ensure that the safety and wellbeing of the patient is protected and that the integrity of the data is preserved.

### 2.2 Study protocol and evaluation criteria

The China Stroke Primary Prevention Trial was a multicommunity, randomized, double-blind clinical trial conducted between May 19, 2008, and August 24, 2013, in 32 communities in the Jiangsu and Anhui provinces of China. The trial consisted of 3 stages: a screening and recruitment period, a 3-week run-in treatment period, and a 5-year randomized treatment period. The 17,312 eligible participants with baseline electrocardiogram measures were stratified according to the presence and/or absence of LVH (the LVH group and the non-LVH group) using the Sokolow–Lyon criteria.^[9]^ Standard 12-lead electrocardiography was routinely performed at baseline and again after 5 years. Electrocardiography was conducted with the patient in a supine position at rest, using electrocardiogram devices routinely used by the institutes. Electrocardiograms were recorded with a calibration mark of 10 mm/mV (or 5 mm/mV) and a paper speed of 25 mm/s, marked on each trace, against which standardized measurements could be made. All electrocardiogram results were read by 2 trained medical practitioners unaware of the participants’ clinical outcome group, and discrepancies were resolved by a third senior medical practitioner.

### 2.3 Criteria for electrocardiogram definitions

The Sokolow–Lyon criteria for LVH were used, and the criteria were defined as follows: SV1 + RV5/6 > 3.8 mV.^[9]^

### 2.4 Blood pressure measurements

Participants were placed in a sitting position with their right arm supported at the level of the heart. Trained volunteers who had been recruited from local medical colleges obtained participant BP measurements using automatic digital sphygmomanometers (Omron HEM 705IT device; Omron-Colin, Japan). The participants were given 3 minutes of rest between each of 3 successive BP readings. The average of the 3 readings was used as the baseline BP value. Hypertension was defined as a SBP of 140 mm Hg or a DBP of 90 mm Hg or higher.

### 2.5 Hypertension control

BP was measured every 3 months during follow-up from 2008 to 2013. The average of 20 measurements at 20 different visits was used as the final BP value. The overall goal of BP control was defined as a SBP of 140 mm Hg and a DBP of 90 mm Hg.

### 2.6 Other definitions

Diabetes mellitus was defined as self-reported clinically diagnosed diabetes, use of hypoglycemic agents, or a fasting blood glucose concentration greater than or equal to 7.0 mmol/L (≥7.0 mmol/L). Participants with an eGFR less than 60 mL/min/1.73 m^2^ and/or proteinuria at baseline were classified as having chronic kidney disease. BMI was calculated as weight in kilograms divided by height in meters squared (m^2^). Serum folate and vitamin B_12_ levels at both the baseline and the exit visits were measured by a commercial laboratory kit using a chemiluminescent immunoassay (New Industrial). Serum homocysteine, fasting lipids, and glucose levels at the baseline and the exit visit were measured using automatic clinical analyzers (Beckman Coulter) at the core laboratory of the National Clinical Research Center for Kidney Disease in Nanfang Hospital, Guangzhou, China.

### 2.7 Statistical analysis

All analyses were performed using EmpowerStats (http://www.empowerstats.com) and the statistical package R. Data are presented as the mean ± standard deviation or proportions. Comparisons between groups were performed using Chi-squared tests for categorical variables and 2-sample *t* tests for continuous variables. Multiple linear and logistic regression analyses were used to assess the associations between baseline ECG LVH and BP control. Values for BP decline after treatment (ΔBP = baseline BP − BP after treatment), relative percentage SBP decline (ΔBP/baseline BP), and unsatisfactory BP control (SBP ≥140 mm Hg or DBP ≥90 mm Hg after treatment) were calculated as indicators of BP control. A 2-tailed *P* value below .05 was considered statistically significant (*P* < .05).

## 3 Results

### 3.1 Study participants and baseline characteristics

As shown in Fig. 1, the present study included 17,312 subjects. A total of 1407 subjects had ECG LVH (age: 61.3 ± 7.6 years, males/females 907/500), and 15,905 subjects did not have ECG LVH (age: 59.7 ± 7.5 years, males/females 6204/9701). The median follow-up period was 4.5 years. The subjects underwent BP measurements every 3 months during follow-up, and the average of BP measurements was recorded as the final follow-up BP. The clinical and demographic characteristics of patients according to the presence or absence of ECG LVH are presented in Table 1. Compared with patients without ECG LVH, patients with LVH were older, had a higher baseline SBP and DBP, were more likely to be male, and were more likely to have lower BMI, total cholesterol, and fasting glucose levels.

**Table 1:**
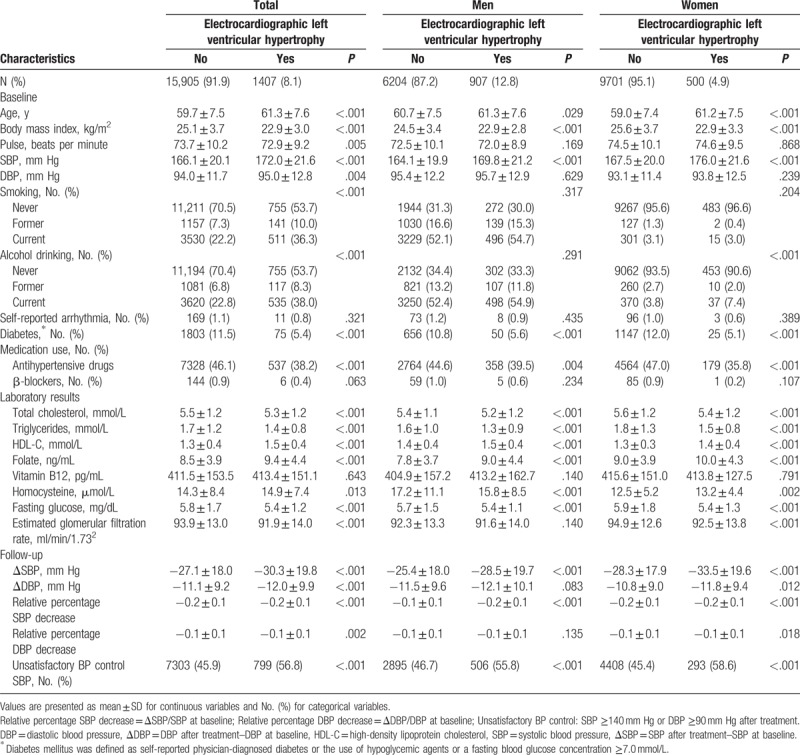
Baseline and follow-up characteristics of the study participants.

### 3.2 Electrocardiographic left ventricular hypertrophy and uncontrolled BP or BP reduction with treatment

The relationship between baseline ECG LVH and failure to achieve BP treatment goals is presented in Table 2. During a median follow-up duration of 4.5 years, the baseline SBP- and DBP-adjusted patients with baseline LVH exhibited significant changes in SBP [β1.34, 95% confidence interval (95% CI) 0.80–1.87, *P* < .0001] and a trend toward significant changes in DBP (β 0.00, 95% CI -0.31 to 0.31, *P* < .9959). In addition, these patients exhibited lower values for the relative percentage decrease in SBP and DBP (β 0.87, 95% CI 0.53–1.20, *P* < .0001) and a trend toward lower values for the relative percentage decrease in DBP (β 0.09, 95% CI -0.26 to 0.43, *P* < .6221). There was significant failure to achieve BP treatment goals in patients with ECG-LVH [odds ratio (OR) 1.33, 95% CI 1.19–1.50, *P* < .0001]. After a further adjustment for baseline SBP and DBP, sex, age, pulse, self-reported arrhythmia, antihypertensive drugs use, β-blocker use, smoking, alcohol consumption, glucose, homocysteine, folate, vitamin B12, total cholesterol, triglycerides, high-density lipoprotein cholesterol, estimated glomerular filtration rate, incidence of failure to achieve BP treatment goals, and changes in SBP and DBP were significantly higher in patients with ECG-LVH, whereas values for the relative percentage decrease in SBP and DBP were significantly lower in patients with ECG-LVH.

**Table 2:**
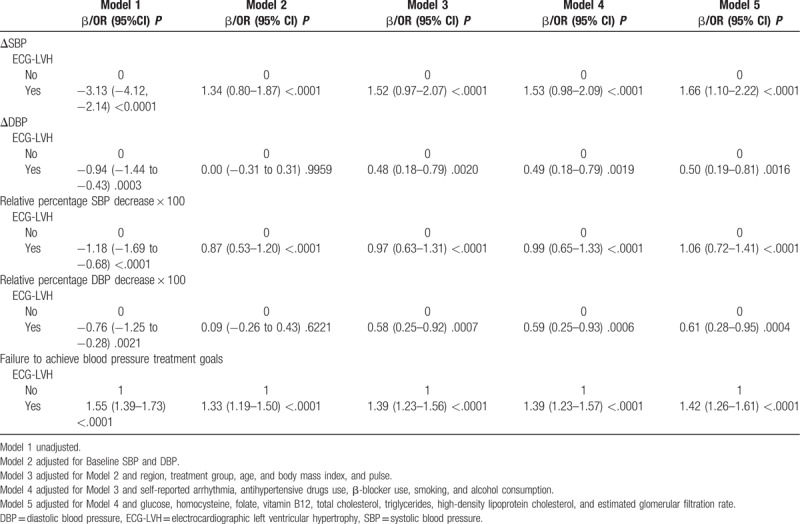
Association between baseline electrocardiographic left ventricular hypertrophy and changes in blood pressure under treatment or failure to achieve blood pressure treatment goals.

### 3.3 Electrocardiographic left ventricular hypertrophy and uncontrolled BP or BP reduction with treatment in men and women

As summarized in Table 3, according to multiple logistic regression models, after adjusting for region, treatment group, self-reported arrhythmia, antihypertensive drug use, β-blocker use, smoking, alcohol consumption, baseline SBP and DBP, sex, age, pulse, BMI, glucose, homocysteine, folate, vitamin B12, total cholesterol, triglycerides, high-density lipoprotein cholesterol, and eGFR, female subjects (OR 1.45, 95% CI 1.18–1.77, *P* < .001) with LVH exhibited higher failure rates for achieving BP goals than male subjects with LVH (OR 1.37, 95% CI 1.17–1.60, *P* < .001). In addition, females with baseline LVH exhibited significantly smaller changes in SBP and DBP (β1.53, 95% CI 0.62–2.43, *P* < .001 and β 0.70, 95% CI 0.22–1.18, *P* < .005, respectively). Males with baseline LVH exhibited significantly smaller changes in SBP (β 1.58, 95% CI 0.87–2.29, *P* < .001); importantly, differences in DBP decreases were not noted in male subjects (β 0.32, 95% CI -0.10 to 0.74, *P* < .132). Similarly, female patients exhibited lower values for the relative percentage decrease in SBP and DBP. Differences in DBP decreases were not noted in male subjects.

**Table 3:**
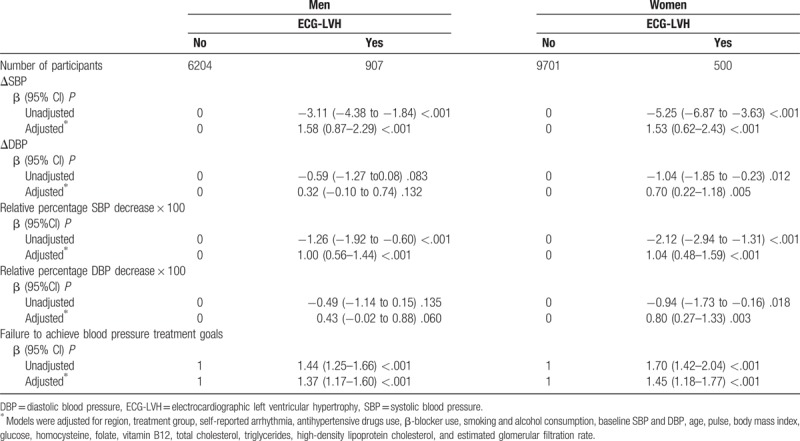
Association between baseline electrocardiographic left ventricular hypertrophy and changes in blood pressure under treatment or failure to achieve blood pressure treatment goals in men and women.

### 3.4 Subgroup analysis

**Figure 2:**
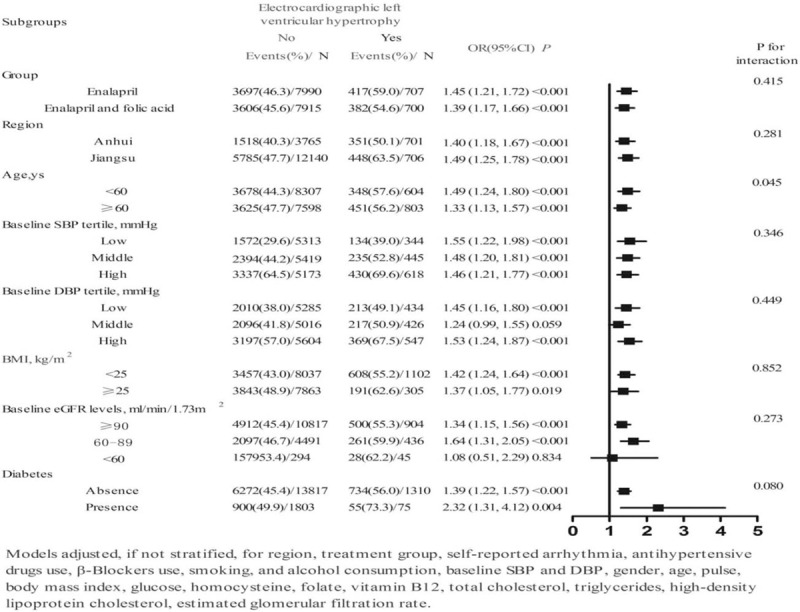
Multivariate logistic regression analysis of the effect of electrocardiographic left ventricular hypertrophy on unsatisfactory blood pressure control in subgroup analyses.

As clinical diversity was observed in the study, we used subgroup analysis to further examine the results. Figure 2 presents the results of the multivariate logistic regression models regarding the assessment of the effect of ECG LVH on unsatisfactory BP control. Following adjustment for the confounders described in Table 2, further observations between the following subgroups were conducted: treatment group (enalapril, enalapril, and folic acid), study center (Anhui, Jiangsu), age (<60, ≥60 years), baseline SBP tertiles (low, middle, high), baseline DBP tertiles (low, middle, high), BMI (<25, ≥25), baseline eGFR levels (≥90, 60–89, <60), and comorbid diabetes (absence, presence). The unsatisfactory BP control rate for these subjects was significantly greater in the presence of baseline ECG LVH. Among these patients, differences were noted in those with comorbid diabetes (OR 2.32, 95% CI 1.31–4.12, *P* = .004). No significant differences were found in patients with baseline eGFR levels lower than 60 mL/min/1.732 m^2^, but the BP control rate was significantly lower in patients with normal BMI (OR 1.42, 95% CI 1.24–1.64, *P* < .001) than in overweight patients (OR 1.37, 95% CI 1.05–1.77, *P* = .019).

## 4 Discussion

A major advantage of the present study is the large sample size derived from the China Stroke Primary Prevention Trial. The China Stroke Primary Prevention Trial was a large randomized trial conducted in adult subjects with hypertension in China without a history of stroke or myocardial infarction. The results of the trial demonstrated that enalapril-folic acid therapy significantly reduced the relative risk of an initial stroke incident (2.7% of participants in the enalapril-folic acid group vs 3.4% in the enalapril alone group) .^[8]^ Given the well-characterized population, the standardized assessment of electrocardiograms using the Sokolow–Lyon criteria, and the 4.5-year longitudinal follow-up period, this study presented a unique opportunity to assess the prevalence of ECG LVH, identify differences between men and women, and explore whether baseline ECG LVH is associated with an increased risk of unsatisfactory BP control in a Chinese hypertensive population. Furthermore, the study provided insight regarding the potential predictive value of unsatisfactory BP control.

The previous study demonstrated that BP, short-term BP variability, and OSA are independently associated with increased left ventricular mass.^[10,11]^ Furthermore, untreated hypertension patients with target organ damage were less likely to receive sufficient antihypertensive treatment.^[12]^ However, the current study demonstrated that ECG LVH in hypertensive patients can be used to identify those at a greater risk of unsatisfactory BP control, notably female patients and patients with comorbid diabetes. However, little is known about the prevalence of ECG LVH in the Asian hypertension population.^[13]^ Our study was the first to report that the prevalence of ECG LVH is 8.1%, and the prevalence is greater in men (12.8%) in a Chinese hypertension population. A nationwide epidemiological survey in Finland determined that the morbidity of ECG LVH was 12.6% in a hypertensive population according to the Sokolow–Lyon voltage criteria,^[14]^ which is higher than the result of our study. Therefore, our study, which is a large randomized multicommunity trial, may better reflect the prevalence of ECG LVH after taking into account differences in lifestyle habits and genetics, and it has been demonstrated from previous studies that ECG has a relatively lower sensitivity than echocardiography in determining LVH. Compared with Caucasians, black individuals have greater precordial QRS voltages. Therefore, many of the LVH criteria have higher sensitivity and lower specificity in detecting LVH in blacks.^[15,16]^ Moreover, geographical differences exist between Asian and European populations. In our study, the prevalence was greater in men (12.8%) than in women. This finding is consistent with those of previous studies.^[17–19]^

As depicted in Table 1, SBP was significantly greater in hypertensive patients with baseline ECG LVH, which was consistent with a previously reported study by Cao et al. ^[20]^ Therefore, ECG LVH could be considered an early indicator of poor BP management. After adjusting for multiplicity, the current study indicated that hypertensive patients with baseline ECG LVH exhibited a higher rate of unsatisfactory BP control (Table 2) and significantly smaller changes in SBP and DBP (Table 2). Lower degrees of relative percentage decreases of SBP and DBP following antihypertensive treatment were further noted (Table 2). The presence of ECG LVH suggests that the left ventricular mass is higher, and the heart ejection fraction is increased. To date, no conclusive data have described the specific pathophysiologic mechanisms of the association between ECG LVH and unsatisfactory BP control. It has been suggested that LVH is a likely consequence of long-standing hypertension, reflecting a poor cardiovascular profile associated with an increased stroke risk.^[21–23]^ It may also be a consequence of mediation by ventricular ectopic beats and ventricular arrhythmias, as ECG LVH is associated with the development of arrhythmia.^[24,25]^ These parameters may increase the risk of unsatisfactory BP control, although further studies are required to test this hypothesis.

Recently, many studies have demonstrated that ECG LVH is significantly associated with cardiovascular events and cerebrovascular incidents in the general population.^[5,17,26,27]^ In the present study, we speculated that unsatisfactory BP control might play an intermediary role in this process. However, the exact mechanism of action remains unclear, and further study is required to address the issue.

In subgroup analyses, the findings of a higher unsatisfactory BP control rate among females or hypertensive patients with diabetes with baseline ECG LVH were compared with those of patients without ECG LVH after adjustment for confounders. The mechanism responsible for the higher risk of unsatisfactory BP control in females with LVH remains uncertain. However, hypertensive females may be more prone to developing higher relative cardiac wall thickness than men.^[28,29]^ Thus, compared with males with similar myocardial mass, females may be more susceptible to impaired myocardial blood flow and unsatisfactory BP control.^[28,29]^ In addition, because males are physically more active than females,^[30]^ increased left ventricular mass may reflect normal physiological adaptation to exercise rather than adverse remodeling more often found in males than in females. Furthermore, high blood glucose levels increase the risk of unsatisfactory BP control, presumably via the association with cardiovascular target organ damage. The Framingham Study cohort ^[31]^ demonstrated that hypertension and diabetes are cardiovascular risk factors responsible for the development of new-onset atrial fibrillation. However, in our study, the unsatisfactory BP control rate of patients with a baseline eGFR of less than 60 mL/min/1.732 m^2^ (<60) was not significant. Several studies have shown that LVH is common in patients with end-stage renal disease,^[32]^ which is an independent predictor of cardiovascular disease and mortality.^[33–35]^ In several studies, LVH was also an independent predictor of cardiovascular disease mortality and heart failure in patients with ≥stage 3 chronic kidney disease.^[36–38]^ In these patients, chronic kidney disease was a greater risk factor for poor BP control than ECG LVH. We could not address the relationship between ECG LVH and BP control in chronic kidney disease patients due to the small sample size. In addition, the risk of unsatisfactory BP control was significantly higher in normal-weight than in overweight patients. Several studies have reported that LVH confers an increased risk of cardiovascular events in overweight patients.^[39,40]^ Even in genetic heart disease, such as hypertrophic cardiomyopathy,^[41]^ obesity is independently associated with increased left ventricular mass and may dictate the progression of heart failure symptoms. In these patients, obesity was a greater risk factor for poor BP control than ECG LVH. This result is different from those obtained in the present study; we could not address the relationship between ECG LVH and BP control in overweight patients due to the small sample size.

Some study limitations should also be taken into consideration. One of the limitations encountered was the single measurement of cardiac activity by electrocardiogram during the baseline physical examination, preventing the exclusion of subsequent abnormalities during the follow-up period. Second, Sokolow–Lyon may be less sensitive in women, and the results may vary with Cornell Voltage criterion. However, we utilized optimal control methods for the LVH criteria. Third, BP measurements were not performed at trough for patients who received antihypertensive drugs, and consequently, the assessment of BP control may have been influenced to a certain extent. In the future, ambulatory BP monitoring may be a favorable method for BP management and evaluation.

We conclude that ECG LVH appears to be an independent predictive risk factor for poor BP control, which suggests that hypertensive patients with LVH have more difficulty achieving target BP and maintaining clinical follow-up, especially female patients and patients with comorbid diabetes.

## Acknowledgment

The authors appreciate the participants who volunteered to participate in the study and the data collection staff of the CSPPT team.

## Author contributions

**Conceptualization:** Xiao Huang.

**Data curation:** Fangfang Fan.

**Formal analysis:** Yan Zhao, Juxiang Li.

**Funding acquisition:** Jing Chen.

**Investigation:** Kui Hong.

**Methodology:** Ping Li.

**Project administration:** Yanqing Wu.

**Resources:** Qinghua Wu.

**Software:** Binyan Wang.

**Supervision:** Xiping Xu.

**Validation:** Yigang Li.

**Visualization:** Xiaoshu Cheng.

**Writing – original draft:** Zhen Wang, Chunyan Zhang.

**Writing – review & editing:** Huihui Bao, Yong Huo, Xiaoshu Cheng.
